# Pediatric Stroke: Overview and Recent Updates

**DOI:** 10.14336/AD.2021.0219

**Published:** 2021-07-01

**Authors:** Mary Hollist, Katherine Au, Larry Morgan, Padmashri A Shetty, Riddhi Rane, Abraham Hollist, Angela Amaniampong, Batool F Kirmani

**Affiliations:** ^1^Memorial Healthcare Institute for Neurosciences, Owosso MI, USA.; ^2^George Washington University, School of Medicine & Health Sciences, Washington DC, USA.; ^3^Bronson Neuroscience Center, Kalamazoo, MI, USA.; ^4^Ramaiah Medical College, M. S. Ramaiah Nagar, Bengaluru, Karnataka, India.; ^5^Optimal Health Medical center, Miami Gardens, FL, USA.; ^6^Indiana University School of Medicine, Indianapolis, IN, USA.; ^7^Texas A&M University College of Medicine, College Station, TX, USA.; ^8^Endovascular Therapy & Interventional Stroke Program, Department of Neurology, CHI St. Joseph Health, Bryan, TX, USA.

**Keywords:** pediatric stroke, intravenous tissue plasminogen activator, Moyamoya, mechanical thrombectomy, Sickle cell disease

## Abstract

Stroke can occur at any age or stage in life. Although it is commonly thought of as a disease amongst the elderly, it is important to highlight the fact that it also affects infants and children. In both populations, strokes have a high rate of morbidity and mortality. Arguably, it is more detrimental in the pediatric population given the occurrence at a younger age and therefore, a longer duration of disability, potentially over the entire lifespan. The high rate of morbidity and mortality in pediatrics is attributed to significant delays in diagnosis, as well as misdiagnosis. Acute stroke management is time dependent. Patients who receive acute treatment with either intravenous (IV) tissue plasminogen activator (tPA) or mechanical thrombectomy, have improved mortality and functional outcomes. Additionally, the earlier treatment is initiated, the higher the likelihood of preserving penumbra, restoring cerebral blood flow and potentially reversing symptoms, thereby limiting disability. Prompt identification is essential as it leads to improved patient care in such a narrow therapeutic window. It enhances the care received during hospitalization and reduces the risk of early stroke recurrence. Despite limited data and lack of large randomized clinical trials in pediatrics, both IV tPA and mechanical thrombectomy have been successfully used. Bridging the gap of acute stroke management in the pediatric population is an essential part of minimizing adverse outcomes. In this review, we discuss the epidemiology of pediatric stroke, the diverse etiologies, presentation as well as both acute and preventative management.

## 1. Introduction

Pediatric stroke is a rare entity. It is often diagnosed with significant delay [1]. Consequently, pediatric stroke is often associated with substantial cost and long-term financial burden on families [2]. Acute neurological changes in the pediatric population have a wide differential diagnosis and various clinical presentations. The lack of familiarity with such as well as its rarity, results in delayed diagnosis, and misdiagnosis. To date, there are limited randomized clinical trials on acute stroke management in the pediatric population. The clinical care directed towards pediatric stroke has been extrapolated from data and experiences with the adult population. Thrombolytic therapy and mechanical thrombectomy are mainly conducted on a case-by-case basis. Nevertheless, their effectiveness has been demonstrated in case reports and series. Understanding of the various clinical presentations of stroke in the young while being cognizant that most pediatrics will lack traditional vascular risk factors frequently encountered in adults is fundamental.

## 2. Epidemiology of Pediatric Strokes

Pediatric stroke is uncommon. Most epidemiological studies are based on relatively few incident cases and lack power to assess for sociodemographic or socioeconomic differences [1]. Therefore, the epidemiological data in children are very different from adults. Furthermore, one of the primary limitations of national database studies is the heterogeneity of data from different centers. Stroke is more difficult to identify due to its varying signs and symptoms. These variations lead to ambiguities in consistency of diagnosis and variability in care.

The incidence of childhood stroke has varied widely in literature. It is estimated that the incidence of strokes in children ranges anywhere from 2.5-13 per 100,000 per year [2,3]. The Canadian Pediatric Ischemic Stroke Registry, a 16-year prospective national-based study provided robust data with documentation of disease incidence, presentation, risk factors, and treatments of pediatric arterial ischemic stroke. Among the 1,129 children enrolled in the study, the incidence of stroke in children aged 29 days to 18 years was 1.72/100,000 per year, and neonates from birth to 28 days was 10.2/100,000 live births [4].

Additionally, boys have a higher prevalence and incidence than girls, and black children are at a higher risk than Caucasian and Asian children [1,4]. The mortality rate of strokes in children is roughly 10-25% [1]. Importantly, hemorrhagic stroke has a significantly higher mortality rate than ischemic stroke. Also, the prevalence of hemorrhagic stroke is approximately 2-fold higher in developing countries [1]. On the other hand, the prevalence of ischemic stroke is historically 4- to 5-fold higher in developing countries, when compared to developed countries. Following the first event, up to 25% of children will have a recurrent stroke [1].

## 3. Clinical Presentation of Stroke in Pediatrics

The structural anatomy of the pediatric brain is remarkably similar to that of an adult, however, there are a multitude of physiologic differences that can result in significant heterogeneity with regards to stroke presentation. Cerebral hemodynamics does not resemble that of an adult until age eight. Prior to this point, pediatric brains are more metabolically active, utilizing up to 200% more glucose than that of an adults’ brain at age five. The increased demand of cerebral blood flow makes them more susceptible to focal neurologic injury during hypoglycemic episodes [5].

DeVeber et al. showed that the most common presenting symptom of strokes in neonates was seizure [4]. In older children, focal deficits (primarily hemiparesis) were more common [4]. It’s important to note that seizures were also quite common among all ages, occurring in 37% of participants in the registry. Overall, non-specific systemic symptoms such as cardio-pulmonary dysfunction, headache, nausea/vomiting, and fever occurred frequently either with or without focal deficits. Non-specific symptoms occurred in 49% of non-neonates and were most prominent with hemorrhagic strokes [4]. As alluded to above, delays in diagnosis are common. In a study conducted by Brauen et al. regarding the diagnostic pitfalls in pediatric ischemic stroke, a correct diagnosis was not obtained in 19 out of 45 pediatric patients with a delay ranging from 15 hours to 3 months from initial presentation [6]. In addition, there also appears to be a significant delay with patients presenting for medical evaluation [7]. Not surprising, given what we know of other health disparities, children of color are more likely to suffer from strokes despite adequate control of risk factors specific to them [8].

Children recover better from neurologic insults than adults, including ischemic and hemorrhagic strokes. While children with strokes have better prognosis than adults, over 20% are moderately to severely neurologically impaired and they have an incidence of depression that is nearly twice as high as their matched cohorts [9]. The lifetime burden of those with residual deficits, particularly if they result in disability is enormous. It may also place them at additional risk of orthopedic complications and fall related injuries [4]. It is important to note that of those who presented with focal deficits, at the time of discharge had residual focal deficits; 70% of non-neonates and 60% of neonates. This is not dissimilar to adult data [4].

## 4. Etiology

Several conditions are associated with pediatric strokes [1]. The most relevant risk factors for the occurrence of stroke in children are vasculopathies, infections, cardiac causes, and coagulopathies [10]. Other risk factors include but are not limited to hematological diseases, renal diseases, child abuse, autoimmune diseases, metabolic disorders, and head trauma [11]. With that being said, no single risk factor results in less neurological harm.

### 4.1 Cardiac Causes

Cerebrovascular diseases in the pediatric population are not as rare as were previously thought. The various types of cerebrovascular diseases along with their frequency and association with stroke have made them the subject of many studies in recent years. The risk factors for cardiac diseases in children range from genetic disorders such as Fabry’s disease, to infections such as meningitis. These risk factors increase the likelihood of cardiac conditions like cardio-embolic strokes, which account for the majority of ischemic strokes in children [10]. In addition to congenital heart defects, the pediatric population is susceptible to the same cardiac abnormalities known to present as risk factors for stroke in adults. These etiologies include bacterial and non-bacterial thrombotic endocarditis, cardiomyopathies, rheumatic heart disease and other valvulopathies.

The prevalence of patent foramen ovale (PFO) is approximately one quarter of the population and in conjunction with underlying hypercoagulable states, can lead to paradoxical embolism [11]. The prevalence of PFO in cryptogenic stroke patients is approximately 40%. It rises to about 55% in cryptogenic patients under the age of 55. The Risk of Paradoxical Embolism (RoPE) study aimed to create a predictive model to determine the probability of an initial stroke being due to PFO. Many people with PFOs remain asymptomatic and require no intervention. However, those with a high RoPE score were found to be at greater risk for stroke and PFO closure was suggested in those cases. While the RoPE score is not the ultimate deciding factor regarding PFO management, it is highly validated in determining the extent of the causal relationship between PFOs and stroke [11].

### 4.2 Sickle Cell Disease

Sickle cell disease (SCD) is a hereditary red blood cell disorder which affects persons of Middle Eastern, South Asian, African, and Mediterranean descent. The occurrence of two abnormally folded hemoglobin molecules results in sickle hemoglobin or hemoglobin S (HbS) which causes sickle cell anemia, the most common and severe form of SCD [12]. Sickle cells become problematic in certain states such as dehydration or acidosis as they can adhere to the vessel walls and impede blood flow, resulting in stroke [13]. In addition, sickled hemoglobin distorts the cell membrane, which causes ion imbalances and facilitates occlusion of vessels. Large vessel disease involving the anterior circulation is the leading cause of symptomatic stroke [14]. Furthermore, patients with SCD have a higher concentration of interleukins, chemokines, and cytokines due to chronic inflammation. Also, increased expression of adhesion molecules reinforces the attachment of sickled RBCs to the endothelium, and increased levels of thrombin along with reduced levels of protein S and C produce a hypercoagulable state [14]. The cerebral metabolic rate of oxygen utilization has also been correlated to ischemia in SCD. The cerebral metabolic rate of oxygen utilization is the product of cerebral blood flow (CBF), arterial oxygen content (CaO_2_), and oxygen extraction fraction (OEF) [15]. In SCD, anemia, hypoxia, and altered oxygen delivery of hemoglobin S (Hb S) can lead to a decrease in the arterial oxygen content. When the cerebral metabolic rate of oxygen utilization does not meet the metabolic demands and falls below the tissue viability threshold, ischemia ensues [15]. Vasculopathies are also frequently associated with an increased risk of ischemia in SCD [16].

The prevalence of stroke is ~4% in patients with SCD. The risk of stroke is most significant during the first decade of life, it drops in the second decade, and rises again in the 3rd decade. Hemorrhagic strokes in the setting of sickle cell disease are more prevalent in adulthood [13]. The risk of ischemic stroke, hemorrhagic stroke, and clinically silent infarcts are all increased in SCD. The other most consistent risk factors for ischemic stroke in children with SCD include a history of transient ischemic attack, acute chest syndrome, degree of anemia, and elevated systolic blood pressure [14]. Sickle cell beta-thalassemia (Hb S/β Th), a subtype of SCD, has the lowest stroke incidence.

### 4.3 Other Causes

#### 4.3.1 Prothrombotic States

Although prothrombotic states are more commonly associated with venous clots, it is an important cause of stroke. This accounts for both ischemic and hemorrhagic strokes. Up to 20 to 50% of children with strokes have an underlying thrombotic disorder; either hereditary or acquired [17]. Antiphospholipid antibodies, deficiency of protein C, MTHFR C677T polymorphism, factor V Leiden mutations, deficiency of antithrombin III, factor II G20210A, and elevated levels of lipoprotein(a), are examples of traits found to be significantly implicated in first occurrences of pediatric arterial ischemic strokes [18]. This also holds true for combined thrombophilias. Obtaining a family history regarding history of venous clots and strokes is essential. Also, a thorough review of medication list can aid in determining the underlying etiology. For example, valproic acid, an antiepileptic, has been linked to acquired protein C deficiency [17].

#### 4.3.2 Vascular

Hemorrhagic strokes in childhood often occur in the setting of vascular malformations. Such malformations are typically due to genetic disorders or are congenital in nature like arteriovenous malformations (AVMs). AVMS are the most commonly encountered and are seen in setting of neurocutaneous syndromes like Struge-Weber disease, Wyburn-Mason Syndrome, PHACE syndrome, hereditary hemorrhagic telangiectasia, and incontinentia pigmenti [17,19]. Arteriopathies are also a common cause of stroke. Arteriopathies can occur in the setting of infections and also with noninflammatory conditions like dissections and fibromuscular dysplasia as well as with moyamoya disease [19].

#### 4.3.3 Dissections

Dissections result in a tear of the arterial wall. It commonly occurs in the setting of trauma or with sudden extension or flexion of the neck such as those that occur with sports. Other mechanisms include coughing, sneezing and arteriopathies, as those seen in Marfan’s syndrome, pseudoxanthoma elasticum, moyamoya disease and fibromuscular dysplasia. Dissections can lead to strokes through the development of an intramural hematoma with subsequent occlusion of the true lumen or via thromboembolism. Symptoms can be delayed anywhere from a day to a week. Compared to adults, intracranial dissections, in particular, those involving the anterior circulation are more common in pediatrics. Also, the incident of childhood dissections is higher in boys when compared to girls [20].

#### 4.3.4 Cerebral Venous Thrombosis

Cerebral venous thrombosis (CVT) in children has been linked to thrombophilias/prothrombotic states including cancer, inflammatory diseases, hematologic conditions and adolescents on oral contraception [21]. Craniofacial infections such as otitis media, sinusitis and periorbital infections are sources for CVT [17]. In the perinatal period, dehydration and perinatal complications are common causes. In the Canadian Pediatric Ischemic Stroke Group Study, the incidence of CVT was higher in neonates when compared to older children [21].

#### 4.3.5 Infections

A causal relationship exists between infectious processes and stroke; this includes bacterial, parasitic, viral, and fungal infections. Focal arteriopathy, vasculitis and or in situ thrombosis are common mechanisms for ischemia. These are often seen in cases of meningitis, encephalitis as well as with HIV and herpesviruses. Other common mechanisms occur either through direct invasion, prothrombotic state, accelerated atherogenesis, enhanced platelet aggregation, or secondary to an inflammatory response. Also, cardioembolic strokes can arise from endocarditis or with heart failure, for example, with Trypanosoma cruzi [21]. Subcortical strokes, in particular those involving the basal ganglia are associated with the Varicella Zoster Virus (VZV) [17]. In addition to HIV and VZV other organisms associated with strokes are: mycoplasma tuberculosis, influenza A, treponema pallidum, Parvovirus B19, and enterovirus, chlamydia pneumoniae, and aspergillus [21].

#### 4.3.6 Oncologic

Strokes can occur in the setting of cancer itself, or as a consequence of treatment, either through radiation or via chemotherapy. Patients with leukemia, and those with radiation therapy, have been cited as the most at risk [22,23]. Most strokes occur early during cancer treatment with a median time of 5 months after cancer diagnosis [24]. Children with cancer have the highest lifetime risk of fatal strokes and the risk is partially due to the exposure of radiation which causes strokes even decades after treatment [24,25]. The risk has been shown to be dose dependent [25]. In a study conducted by Haddy et al., radiation dose to the brain was significantly associated with long-term cerebrovascular mortality among 5-year survivors [26]. Sun et al. evaluated the relationship between cancer and childhood ischemic strokes from children enrolled in the International Pediatric Stroke Study between January 2003 and June 2019 and found that cancer was present in 3.3% of children with AIS and in 10.7% with cerebral sinovenous thrombosis [27]. These findings are consistent with the findings of other published studies, with hemorrhagic and ischemic stroke occurring at similar frequencies [22, 28].

In regards to chemotherapy, Cytarabine and L-arginase are highly associated with increased risk of stroke [19].

#### 4.3.7 Genetics/Hereditary Conditions

Genetic disorders are an important risk factor for strokes in the pediatric population. Obtaining an adequate family history and well as performing a thorough physical examination are essential for identification of the underlying genetic disorder.

Common genetic causes of strokes are listed in Table 1. Other genetic mutations associated with strokes are neurofibromatosis Type 1 and hereditary hemorrhagic telangiectasia. Mutations involving cystathionine beta synthase and methylenetetrahydrofolate reductase result in hyperhomocysteinemia and homocysteinemia. Hyperhomocysteinemia can also arise from dietary deficiencies. Deficiencies in cyanocobalamin, pyridoxine or folate causes premature atherosclerosis and vessel wall injury [29-37].

### 4.4 Moyamoya Disease

Moyamoya Disease (MMD) is a non-inflammatory vasculopathy that is associated with the stenosis of intracranial arteries, notably the distal internal carotid artery (ICA) and its branches. Patients with Moyamoya disease are likely to have a stroke due to the occlusion and stenosis of the blood vessels. Compensatory mechanisms allow for the formation of collateral vessels at the base of the brain. Moyamoya has a variable presentation that can be broadly categorized into two groups; symptoms due to ischemia or symptoms due to intracranial hemorrhage which occurs as a complication of the collateral blood vessel network. Although most patients present with ischemia, adults are more likely to present with hemorrhagic strokes than children. Moyamoya has been linked to both genetic and environmental factors and should be a consideration for any patient displaying neurological deficits or cerebral ischemia. Moyamoya can be confirmed via various radiographic studies including CTs, MRIs, and angiography [38].

**Table 1:** Disorders associated with Pediatric Stroke.

	Gene	Mode of inheritance	Clinical Features	Vascular complications
**Marfan Syndrome**	Chromosome 15q, FBN1	Autosomal dominant	Ectopic lens, arachnodactyly, aortic dilatations, mitral-valve prolapse, familial aortic aneurysms and dissections, skin and skeletal anomalies- pectus carinatum, pectus excavatum, scoliosis and pes planus	Arterial dissections (vertebral, carotid and aorta)
**Pseudoxanthoma** **Elasticum**	Chromosome 16p 13.1, ABCC6	Autosomal recessive	Skin changes	Intracranial aneurysm, dissections, moyamoya -like vasculopathy, small vessel disease
**Ehlers-Danlos Syndrome- type IV (mainly associated with vascular complications)**	Chromosome 2, COL3A1	Autosomal dominant	Hyperextensible joints, easy bruising skin, vascular lesion	Aneurysm, carotid-cavernous fistulae, dissections
**Progeria**	Chromosome 1q21.2, LMNA Gene	Autosomal dominant	Premature aging; alopecia lipoatrophy, atherosclerosis, joint contractures. Coronary artery disease, skeletal abnormalities such as mandibuloacral dysplasia	Cardiovascular disease, stroke- involving large intracranial arteries, vertebral & carotid arteries
**Mitochondrial encephalopathy, lactic acidosis, and stroke-like episodes (MELAS)**	mitochondrial DNA; A3243G gene (most common mutation)	Maternal inheritance	Developmental delay, recurrent vomiting, headaches, deafness, muscle weakness. diabetes mellitus, renal disease, short stature, cardiomyopathy	Recurrent stroke-like episodes involving non-vascular territories
**Fabry Disease**	Alpha Galactosidase	X-linked	Dysmorphic features, anhidrosis, angiokeratomas, acroparesthesia, ophthalmologic complications	Small vessel ischemic disease, intracranial arterial dolichoectasia, intracerebral hemorrhage, cardiogenic embolism
**Von Hippel-Lindau Disease**	Chromosome 3p25-26, VHL tumor suppressor gene	Autosomal dominant	Pheochromocytomas, renal cell carcinoma, pancreatic cystadenomas, hemangioblastomas (CNS & retinal)	Intracranial hemorrhage, aneurysm
**Sturge- Weber Syndrome**	GNAQ gene	Somatic mosaic R183Q mutation	Port-wine stain, glaucoma, mental retardation, seizures, contralateral hemiparesis & hemiatrophy	Intracranial hemorrhage

## 5. Imaging and Diagnostic Testing

### 5.1 Use of CT, CTA and MRI and Management Strategies

Initial management should focus on early detection and correctly diagnosing the type of stroke, along with immediate stabilization and resuscitation. Correction of underlying conditions such as hypoxia, febrility, hypoglycemia, or significant metabolic derangements are imperative.[Fig F1-ad-12-4-1043]

**Figure 1. F1-ad-12-4-1043:**
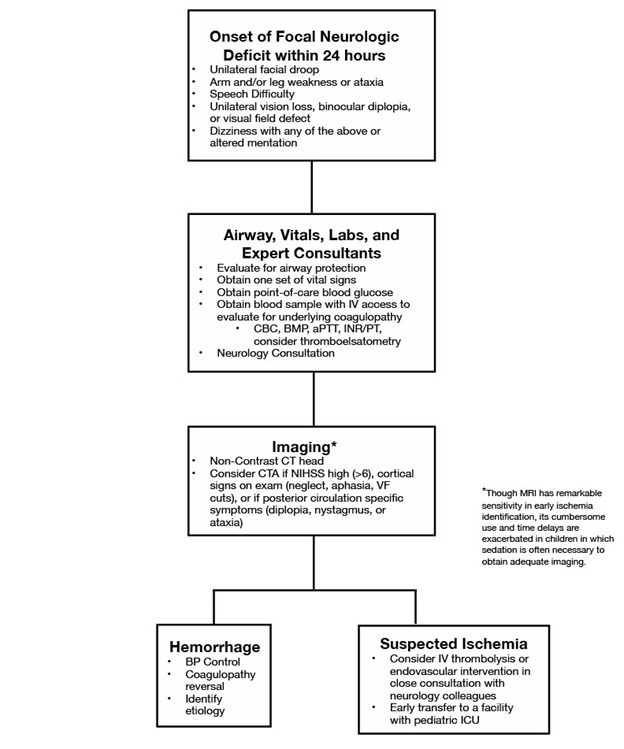
Management of pediatric stroke flowchart.

As mentioned, most children have no previously known risk factors for acute ischemic stroke. A CT scan in the acute setting is often non-diagnostic. Only 50% of non-contrast CT scans obtained at presentation revealed any findings consistent with evolving ischemic changes [39]. Despite this, CT imaging remains the modality of choice for acute presentations of focal neurologic deficits. It has a very high sensitivity for identifying acute hemorrhage and can also assist the clinician in rapidly narrowing the differential diagnosis [40]. Early ischemic infarcts may show a hypodense area corresponding to a vascular territory or other early changes such as sulcal effacement or loss of gray-white differentiation [40]. Radiation effects are cumulative in nature and therefore more concerning when used earlier in life. Still, it is clear the diagnostic information provided far outweighs the risks; especially with those presenting with focal neurologic deficits or otherwise unexplained depressed levels of consciousness.

In regards to advanced imaging, due to the altered hemodynamics of pediatric patients, particularly in those less than 8 years old, CT perfusion has not been validated with the same parameters for adults and should not be relied upon as the basis for mechanical thrombectomy in an extended time window. Also, there is limited evidence for the use of the modified ASPECTS (The Alberta Stroke Program Early CT Score) scoring system. However, it could serve as an additional data point to assist in decision making for mechanical thrombectomy in the extended time window [43].

In the event of a suspected large vessel occlusion, CT angiography should be considered.

Though few clinicians have extensive experience in mechanical thrombectomy and intra-arterial management strategies for acute ischemic stroke, these should be considered on a case-by-case basis when a large vessel occlusion is identified. Transfer to a facility with a neuroendovascular specialist who is skilled with pediatric endovascular techniques should be considered on a case-by-case basis.

For hemorrhagic strokes, nicardipine is the medication of choice for blood pressure management. It has a short half-life and is easily titrated to desired endpoints. There are clearly established blood pressure goals, however, normal blood pressures in pediatrics are lower than in adults. This should be considered when determining blood pressure targets. Similar to adults, reversal of known coagulopathies is recommended [44].

## 6. Acute and Preventative Management

### 6.1 Intravenous Tissue Plasminogen Activator (tPA) and Mechanical Thrombectomy

#### 6.1.1 Intravenous Tissue Plasminogen Activator (tPA)

The most comprehensive study to date regarding stroke and tPA uses in pediatrics is the TIPS study which was published in 2010. The TIPS study was an international multicenter, dose-adaptive, phase 1 study [42]. Children aged 2 through 17 years who presented with AIS were eligible for enrollment to receive IV tPA if initiated within 4.5 hours of stroke onset [42]. Three dosing tiers were planned (0.75, 0.9, and 1.0 mg/kg of IV tPA), given over the period of one hour [42]. The purpose of the TIPS study was to define safety criteria to guide the use of tPA in pediatric patients. However, the study was terminated due poor recruitment.

In a retrospective multicentric study in France conducted between 2012 and 2015, 11 pediatric patients (median age 11.8 years) with arterial ischemic stroke received intravenous recombinant-tPA [44]. The median time from onset to treatment was 240 minutes and the median time from onset to magnetic resonance imaging was 165 minutes [43]. Predominant clinical presentations consisted of acute hemiplegia/hemiparesis and dysphagia, and the median NIHSS score was 10 [43]. Most strokes involved the middle cerebral artery territory. No intracranial or peripheral bleeding was reported after treatment and most had a favorable outcome [45].

These two studies highlight the challenges involved in the management of pediatric stroke. To start, significant variability among facilities in the management of pediatric stroke exists. Furthermore, providers who care for these patients may not be familiar with the use of tPA in the pediatric population. This necessitates a need to standardize pediatric stroke care. In the case of the TIPS study, while an earnest effort was made to establish such guidelines, the fact is that the patient population who would benefit from the treatment is very few as reflected by its poor recruitment. Capturing this already small population and enrolling them in comprehensive studies like TIPS is very challenging due to the time-sensitive nature of the treatment. Although the study was ultimately terminated, the TIPS trial helped to inform and optimize acute pediatric stroke care. Furthermore, the trial served as a catalyst for significant changes in participating centers; this is demonstrated in data provided from subsequent retrospective studies as previously described.

Unfortunately, no consensus on specific inclusion criteria for the use of IV thrombolytics in the pediatric population exists. Several recommendations on dosing variations and timing have been made based on the understanding of certain differences in relative amounts of clotting factors and fibrinogen levels [46]. Dosing variations of 0.3mg/kg to 1.0mg/kg has been recommended by various societal groups, without any adequate randomized data [42]. It is the author’s opinion that strict adherence to adult guidelines regarding contraindications for IV thrombolysis be followed in the absence of data in the pediatric population.

Given the difficulties in regards to early recognition, decision-making and management, it is critical to have a pediatric stroke pathway with multidisciplinary input to support clinicians.

#### 6.1.2 Mechanical Thrombectomy

Similar to the use of tPA in pediatrics, mechanical thrombectomy in childhood stroke is also not well-established. Likewise, there are several small retrospective studies investigating its feasibility and efficacy. These studies have demonstrated its safety and benefit with long-term neurological outcomes in selected pediatric patients.

In a comprehensive review of available literature by Satti et al., 29 pediatric patients who had undergone ischemic stroke treatment using modern devices (excluding older techniques such as wire manipulation and balloon angioplasty) between 2008 and 2015 were identified [47]. The average age was 10.3 years, ranging from 1.8 to 18 years, with an average pediatric stroke scale score of 18.1 [47]. There was a broad range of reported time to treatment, with several cases of extremely delayed recanalization (overall range of 1.5 - 120 hours). Excluding these outliers, the average time to treatment was 8.8 hours [47]. This excess lag time compared to adult trials is likely due to delay in recognition of acute stroke in children, as well as a lack of standardization of neurointerventional care. A variety of treatments were recorded, including stent retrievers, the Penumbra system, and other mechanical devices including Merci, Solitaire, Trevo, and Wingspan [47]. Given the severity of stroke on presentation, there were relatively few adverse events reported. Most adverse effects were from patients with basilar occlusion associated with high baseline pediatric NIHSS (National Institute of Health Stroke Scale) scores [47]. These events included severe cerebellar edema and hydrocephalus, asymptomatic hemorrhage, and basilar vasospasm. Of the 29 patients included in the study, clinical outcome was reported in 23 patients, 20 of which achieved favorable clinical outcomes with a mean mRS (modified Rankin Scale) score of 0-1 [47].

Similar outcomes like those seen by Satti et al. are also echoed by subsequent systematic reviews and meta-analyses conducted by Bhatia et al. and Fragata et al. These systematic reviews and meta-analyses investigated the efficacy of mechanical thrombectomy for pediatric ischemic stroke due to large vessel occlusion [48,49]. The former study found that mechanical thrombectomy resulted in good long-term neurological outcomes (mRS scores between 0-2) in 87 out of 96 cases, good short-term outcomes (reduction in NIHSS by ≥ 8 points) in 55 out of 79 cases, and successful recanalization (modified Treatment in Cerebral Ischemia, mTICI, 2b/3) in 86 out of 98 cases [48]. Death occurred in two patients and symptomatic intracranial hemorrhage in one patient [48]. The latter study by Fragata et al. reported seven cases over a period of seven years, in which five patients had cardiac disease, two of which were under external cardiac assistance, with a median time from onset of symptoms to treatment being approximately 7 hours [49]. Mechanical thrombectomy was performed using a stentriever in 3 patients, aspiration in 3 patients, and a combined technique in one patient [49]. While six patients had good recanalization, two patients died, one after hemorrhagic transformation of a middle cerebral artery infarct, and the other due to extensive brainstem ischemia caused by varicella vasculitis [49]. In a slightly larger retrospective study, which included nineteen patients who underwent endovascular thrombectomy between 2008 and 2017 with an average age of 10.9 years and a NIHSS score of 13.9, a transfemoral approach utilizing stent retrievers and aspiration resulted in successful revascularization in 89.5% of patients with an average of 2.2 passes and a recanalization time of 48.7 minutes [50]. Upon discharge the average reduction in NIHSS was 10.2 [50]. Although one patient had post-revascularization seizure, no other complications occurred [50].

Similar to their use in their adult counterparts, the use of modern devices for mechanical thrombectomy in pediatric populations appears to be associated with low complication rates and good clinical outcomes. While the existing literature consists of small cohort studies, case series, and case reports, publications of pediatric cases have been on the rise in recent years, and this has helped guide our decision-making.

### 6.2 Management and Preventative Care in Special Cases

#### 6.2.1 Stroke in Sickle Cell Disease

The hallmark of stroke prevention and treatment in SCD is chronic red blood cell (RBC) transfusion with a target of maintaining total hemoglobin at 10-12.5 g/dL and sickle hemoglobin (HbS) at less than 30%. The Stroke Prevention Trial in Sickle Cell Anemia (STOP 1998) compared RBC transfusion with standard care in patients with abnormal TCD velocities. Transfusion therapy resulted in a 92% risk reduction for stroke in SCD [51,52]. STOP II, a follow-up trial conducted in 2005 demonstrated the importance of continued RBC transfusion therapy in regards to stroke prevention in patients despite normalization of TCD velocities [53]. Most importantly, cessation of therapy was associated with worsening TCD velocities. A subsequent trial also validated similar findings of increased frequency of new brain lesions in patients who stopped transfusion therapy [54,55]. Interestingly, transfusion therapy also decreased the incidence of Acute Chest Syndrome (ACS) in children with a high risk of stroke. Similar to the STOP trial, the Silent Cerebral Infarct Multi-Center Clinical Trial (SIT) which compared RBC transfusion therapy to standard care showed a 6% recurrence of silent cerebral infarcts (SCIs) in the transfusion group as compared to the 14% recurrence in the observation group [56]. Based on the results of the SIT, STOP and STOP II trials, it was determined that chronic RBC transfusion therapy decreased the occurrence of SCIs in children with abnormal TCD velocities. However, the therapy made little or no difference in children with previous evidence of SCIs but with normal TCD velocities [54].

Transfusion therapy can be achieved via exchange transfusion (manual or automated), simple transfusion or with automated red cell exchange. The risks of transfusion include but are not limited to alloimmunization, hyperviscosity, hemolytic transfusion reaction and iron overload. Those treated with chronic transfusion are at highest risk of iron overload, which can be rectified through the addition of chelation therapy [57,58].

Hydroxyurea, an oral medication, has been studied as an alternative to red blood cell transfusion/chelation therapy. Hydroxyurea induces the expression of fetal hemoglobin, which prevents the formation of polymers that ultimately cause the sickling of red blood cells [59]. In studies comparing the administration of hydroxyurea to placebo, there was a statistically significant improvement in pain, a decrease in incidence of acute chest syndrome, and an increase in fetal hemoglobin concentration as well as neutrophil counts [59]. The SWiTCH 2012 (Stroke With Transfusions Changing to Hydroxyurea) and TWiTCH 2016 (TCD With Transfusions Changing to Hydroxyurea), compared the effects of hydroxyurea vs. chronic blood transfusion therapy in patients with sickle cell disease. Both the SWiTCH and TWiTCH trials administered doses of hydroxyurea up to the maximum tolerated dose (MTD). They also received transfusion in the interim. Once MTD was attained, transfusion was discontinued and phlebotomy was initiated with monthly removal of 10 mL/kg of blood for management of iron overload [60,61].

The SWiTCH trial was designed to assess the effects of switching from transfusion therapy to hydroxyurea for secondary stroke prevention in children with the HbSS and HbSβ^0^ genotype and a history of stroke. Patients included in the trial had a prior history of stroke and iron overload while on transfusion therapy. In regards to primary outcome, 10% of those in the hydroxyurea/phlebotomy arm had a recurrent stroke while none occurred in the transfusions/chelation arm. The second primary outcome of liver iron content (LIC) did not significantly differ between both arms. Based on lack of superiority of the LIC in the hydroxyurea/phlebotomy arm, the trial was terminated following the initial interim analysis as it was determined that there was a low likelihood of meeting the composite primary outcome. Therefore, results of the SWiTCH trial were inconclusive. It is unclear if switching to hydroxyurea from transfusion therapy was beneficial. The study concluded that transfusion/chelation should be maintained as the primary therapy for children with SCD, iron overload and a history of stroke [60].

The TWiTCH trial aimed to assess the efficacy of hydroxyurea *vs.* standard transfusion therapy for children with sickle cell anemia and elevated transcranial Doppler (TCD) flow velocities who were at high risk for stroke. The TWiTCH trial was conducted in children from the ages of 4 to 16 with the HbSS and HbSβ^0^ genotype, along with abnormal TCD velocities and a history of RBC transfusion therapy for at least 12 months [61]. It excluded those with severe vasculopathy. No strokes or SCIs were reported in the trial, also, patients who were weaned off chronic transfusion therapy and were maintained on hydroxyurea did not revert back to abnormal TCD velocities. The results showed that hydroxyurea can be used as an alternative for primary prevention of stroke.

Early screenings for children with SCD are imperative for the proper management of SCD as well as its associated complications. Providers must be aware of the prevalence of SCIs in particular, as they normally do not show any clinical symptoms. Due to its association with SCD, the treatment and prevention of cerebro-vascular disease in children with SCD has been the primary focus in many studies in the past two decades. Hydroxyurea is an effective treatment for sickle cell disease as it can ameliorate SCD symptoms by increasing the levels of fetal hemoglobin. However, RBC transfusion therapy is often required in order to treat or prevent disease complications such as SCIs, stroke, and ACS. Given the results of recent studies, there is insufficient data to provide an argument for or against the use of hydroxyurea as a substitute for transfusion therapy in the prevention of primary or secondary SCIs or strokes. Chronic RBC transfusion therapy coupled with chelation therapy is the preferred course of treatment for managing SCIs and stroke in patients with SCD and abnormal TCD velocities.

#### 6.2.2 Stroke in Moyamoya Disease

Currently, there is no known treatment to reverse the progression of Moyamoya. However, therapies do exist to prevent further strokes in symptomatic patients [62].

The current treatment options for MMD are divided into conservative or surgical interventions.

Conservative treatment includes medical interventions with anti-platelets, and symptomatic management such as anti-epileptics, as well as headache prophylaxis. Surgical treatments are divided into direct revascularization and indirect revascularizations [62]. Direct revascularization involves a superficial temporal artery (STA) to middle cerebral artery (MCA) bypass. Indirect revascularization involves placing tissue that is supplied by the external carotid artery onto the brain surface in order to promote angiogenesis [63]. The preferred intervention has been the subject of many recent studies and is still highly debated.

In a meta-analysis performed by Li et al., involving a total of 2,287 participants (1,525 were in the revascularization surgery group, and 762 were in the conservative treatment group), it was determined that surgical intervention was superior to conservative treatment in the prevention of stroke and was associated with improved cerebral perfusion [62]. More specifically, direct bypass surgery was more advantageous at reducing stroke risk than indirect revascularization and also had better angiographic outcomes. Surgical intervention also had a significant advantage in reducing the incidence of deaths due to bleeding as compared to conservative treatment. There was no significant difference with incidence of perioperative complications among the two surgical approaches. In terms of stroke subtype, those who presented with hemorrhages benefited from bypass surgery as it was more efficacious at secondary stroke prevention. However, there was no significant difference between surgical and conservative treatments in reducing stroke risk in patients with ischemic MMD symptoms [62].

Macysyzn et al. conducted an analysis utilizing a decision analytical model which compared surgical techniques that involved direct, indirect, and combined therapies for MMD. Combined therapy is often chosen in an attempt to reduce the risk of further hemorrhage after surgery, although there is little evidence as to whether it actually improves or worsens the outcome. For the pediatric population, it was determined that there was no significant difference between the combined and indirect approach [63,64,65]. However, both were superior to the direct approach. This finding remained at both 5- and 10-year follow up. Interestingly, in the adult population, at a 4 year follow up time interval, indirect revascularization was superior to direct bypass for the treatment of MMD. This was attributed to the increased technical difficulty involved with the direct approach, increased risk of hyperperfusion syndrome, and increased risk of accelerated stenosis all outweighing its benefits as a therapeutic intervention [63].

While the data from both meta-analysis evaluated the optimum treatment for secondary prevention, it is important to highlight the fact that their outcomes were different due to the overall aims of both studies. Li et al. sought to determine what intervention would be the most effective in reducing the incidence of secondary stroke in MMD while Macyszyn et al. aimed to determine the superior intervention in terms of quality-adjusted life years.

Nevertheless, both studies allow for valuable insights regarding MMD in pediatric populations and how we may better intervene and approach its management. Although much remains to be elucidated regarding MMD, the current consensus is that interventions should be technically simple, safe, and prompt. Future research should aim to conduct randomized controlled trials in order to corroborate and confirm the data and results from the current studies, but also, to reduce morbidity and mortality.

## 7. Conclusion

Strokes can occur even in our younger patients, and there is a need for more education on pediatric strokes. Promoting awareness bridges the gap with management comfort amongst pediatricians and most notably, adult neurologists. Pediatric strokes are often challenging to identify due to the subtleness of signs and symptoms, therefore, are frequently undiagnosed or misdiagnosed. Comprehensive assessment of neurologic status, clinical presentation, and radiological imaging is necessary in childhood stroke diagnosis. Timely diagnosis is of the essence. Doing so provides favorable long-term outcomes and minimizes significant neurologic deficits that may emerge as a result of an unmanaged stroke. Collaborative decision-making processes, as well as MRI if able, are recommended to rule out stroke mimics in children. While tPA is a powerful thrombolytic agent used for early treatment in adults, its use for treating infants and young children is not well established due to the lack of clinical trials. In situations where randomized clinical trials are not feasible, systematic data collection gives valuable information that otherwise would not be brought to light. National and international registries are encouraged as small case studies and case series are not sufficient in providing relevant data of treatment safety and efficacy.
